# Anti-Metastatic Effects of *Plukenetia volubilis* (Sacha Inchi) Husk Extract via EGFR and EMT Pathways and Other Antitumor Effects in Colon Cancer

**DOI:** 10.3390/ijms262110514

**Published:** 2025-10-29

**Authors:** Supawadee Osotprasit, Saowaros Suwansa-Ard, Scott F. Cummins, Tianfang Wang, Tepparit Samrit, Athit Chaiwichien, Stuart J. Smith, Narin Changklungmoa, Pornanan Kueakhai

**Affiliations:** 1 Faculty of Allied Health Sciences, Burapha University, Chonburi 20131, Thailand; 63810104@go.buu.ac.th (S.O.); tepparit.sa@go.buu.ac.th (T.S.); 63810103@go.buu.ac.th (A.C.); narinchang@go.buu.ac.th (N.C.); 2 Centre for Bioinnovation, University of the Sunshine Coast, Maroochydore, QLD 4558, Australia; ssuwansa@usc.edu.au (S.S.-A.); scummins@usc.edu.au (S.F.C.); twang@usc.edu.au (T.W.); ssmith16@usc.edu.au (S.J.S.); 3 School of Science, Technology and Engineering, University of the Sunshine Coast, Sippy Downs, QLD 4558, Australia; 4 Food Bioactive Compounds Research Unit, Faculty of Allied Health Sciences, Burapha University, Chonburi 20131, Thailand

**Keywords:** colorectal cancer, sacha inchi, anticancer activity, omics, chemical properties

## Abstract

Colorectal cancer treatment primarily relies on chemotherapy, which often causes significant side effects. Sacha inchi, a plant known in traditional medicine, has shown promise in various therapeutic applications. However, despite its potential, the specific mechanisms remain poorly understood, particularly regarding its husk components. This study investigates sacha inchi husk extract’s chemical properties and its effects on human colorectal cancer cells. GC/MS and LC/MS analyses revealed a rich profile of phenolic and flavonoid compounds, with naringenin and lidocaine as predominant components. The extract demonstrated significant dose-dependent inhibition of colorectal cell migration, invasion, and colony formation while exhibiting no cytotoxicity toward normal colon epithelial cells. Transcriptomic and proteomic analyses showed downregulation of migration- and invasion-related genes in cancer cells, and Western blot analysis confirmed reduced expression of MMP2, MMP9, and N-cadherin. EGFR pathway analysis showed decreased expression of RAS (−0.2-fold), MAK (−0.26-fold), and ERK (−0.54-fold) genes, indicating suppression of epithelial–mesenchymal transition (EMT). These findings demonstrate that sacha inchi husk extract effectively inhibits metastasis in colorectal cancer cells through the upstream (EGFR) and downstream (EMT) pathways, suggesting its potential as a dietary supplement or therapeutic agent for colorectal cancer treatment. Our research provides evidence for the development of natural, less toxic alternatives.

## 1. Introduction

The colon is one of the most common sites for cancer development, and malignant tumors at this site are associated with high mortality rates [1]. In fact, colon cancer is the second leading cause of cancer deaths worldwide, with more than 1.9 million new cases and more than 930,000 deaths worldwide in 2020 [2]. As such, there has been intense research focus on understanding the mechanism of colon cancer development and treatment.

Tumor metastasis is a complex process whereby epithelial cells migrate, invade, and spread from the primary tumor to enter the blood circulation. Initially, this involves the tumor cells passing through the basal membrane and then penetrating the extracellular matrix (ECM) into the blood circulation [3]. Some tumor cells can also transform through blood vessels and cause distant metastasis. Then, colonizing tumor cells adapt to the new microenvironment and switch from migration mode to metastasis mode to generate metastases [4]. The adhesion of tumor cells to the basement membrane causes endothelial contraction [5], as well as tumor cell secretion of proteases, such as matrix metalloproteinases (MMPs) and serine proteinases, that initiate the degradation of the ECM [6]. MMPs can also be secreted by endothelial cells. In response, basement membrane type IV collagen and other matrix proteins are reduced [7]. Therefore, MMPs can be considered a biomarker of invasion, and their suppression is expected to inhibit invasion and metastasis.

Treatment for colon cancer depends on the location of the tumor. The current treatment methods include surgery, radiation therapy, and targeted therapy; however, chemotherapy is the most common form of treatment, with a 20–80% chance of recurrence or not responding to the treatment. Even though chemotherapy drugs destroy all types of cancer cells, normal body cells can also be destroyed, particularly in patients who are already weak. Therefore, chemotherapy causes a high number of side effects that, in turn, increase the risk of death. Thus, alternative treatments, which are safe and have no side effects, are needed for colon cancer. Numerous studies have shown that medicinal herbs contain natural chemicals that effectively treat certain cancers, including colon cancer. Moreover, these may be potentially safer and less toxic compared to conventional therapies [8,9].

Plant-derived active ingredients play an important role in modern biomedical research due to their diverse biological properties, such as antioxidant, anti-inflammatory, and anticancer activities. In particular, in the field of cancer therapy, plant-derived active ingredients can act through multiple biological signaling pathways, such as STAT-3, PI3K/Akt, and EGFR, which results in the inhibition of cell proliferation, induction of apoptosis, and inhibition of the epithelial–mesenchymal transition (EMT) involved in cancer cell metastasis [10]. Furthermore, research has found that prunin and tangeretin, plant flavonoids, have anticancer potential through diverse biological mechanisms. They exhibit antioxidant and anti-inflammatory properties and promote cancer cell death by inducing apoptosis and cell cycle arrest. They can also be used in combination with conventional treatments, such as chemotherapy, to increase efficacy and reduce drug resistance [11,12]. This suggests their potential applications in various chronic diseases and cancers. In addition, biotechnological developments, such as the use of plant-derived ingredients in nanofiber and hydrogel forms, have been carried out for efficient drug delivery and tissue repair [13]. The biodegradability, safety, and environmental friendliness of plant-derived ingredients make them an attractive option for developing new, sustainable, and effective therapeutic approaches in the future.

One particular herb of interest that has been used in the treatment of colon cancer is derived from sacha inchi (*Plukenetia volubilis* Linneo), a woody vine plant within the family Euphorbiaceae. It is commonly grown in many regions of the Peruvian Amazon, where it is native [14]. The plant’s peanuts have significant economic value in the cosmetic, pharmaceutical, and food industries. The global sacha inchi market volume was USD 92 million in 2020 and is expected to gain a higher market share, with a compound annual growth rate of 4.7%, reaching USD 125.8 million during 2021–2027 [15]. Sacha inchi peanuts contain 1.24% total fat, 74.56 mg/g polysaccharides, 69.42 mg/g tannins (an important phenol compound), 0.36 mg/g flavonoids, and 0.11 mg/g free phenolic acid. In addition, sacha inchi seed shells contain higher α-tocopherol than the seeds, while linolenic acid (omega-3) and linoleic acid (omega-6) are found in equal ratios. The unsaturated fatty acids and polyphenols are known to have properties (e.g., antioxidant, antiproliferative, and anti-inflammatory activities) that prevent or reduce the risk of many ailments and diseases, including cancer and obesity [16]. Some studies have tested the benefits of sacha inchi extracts, but not profoundly enough, and the potential active chemicals within sacha inchi husks have not been tested. Therefore, it cannot currently be developed as a drug or health product.

Multi-omics technology has been applied as an important approach for the screening and functional analysis of natural extracts in colon cancer. Recent research has shown that omics-guided screening can reveal the complex molecular mechanisms of the disease and facilitate the discovery of high-potential biomarkers, such as autoantigens and autoantibodies associated with colorectal cancer [17]. Furthermore, organoid models combined with multi-omics analysis can screen natural active compounds and develop personalized drugs [18]. In addition, research has emphasized the role of multiple omics fields, such as genomics, proteomics, and metabolomics, in developing precision screening and therapy, reflecting the trend of integrating biological data to enhance the diagnosis and treatment of colon cancer using natural compounds [19].

In this study, we investigated sacha inchi husks using chemical analysis, followed by extract analysis evaluation against colon cancer cells, using a combination of in vitro bioassays and multi-omics analysis. The outcomes provide evidence for sacha inchi husk extracts having anticancer activity, which may provide abundant raw materials for the increased commercial value of *Plukenetia volubilis* in Thailand.

## 2. Results

### 2.1. Phytochemical Screening of the Husk Extract Using Mass Spectrometry

**Figure 1:**
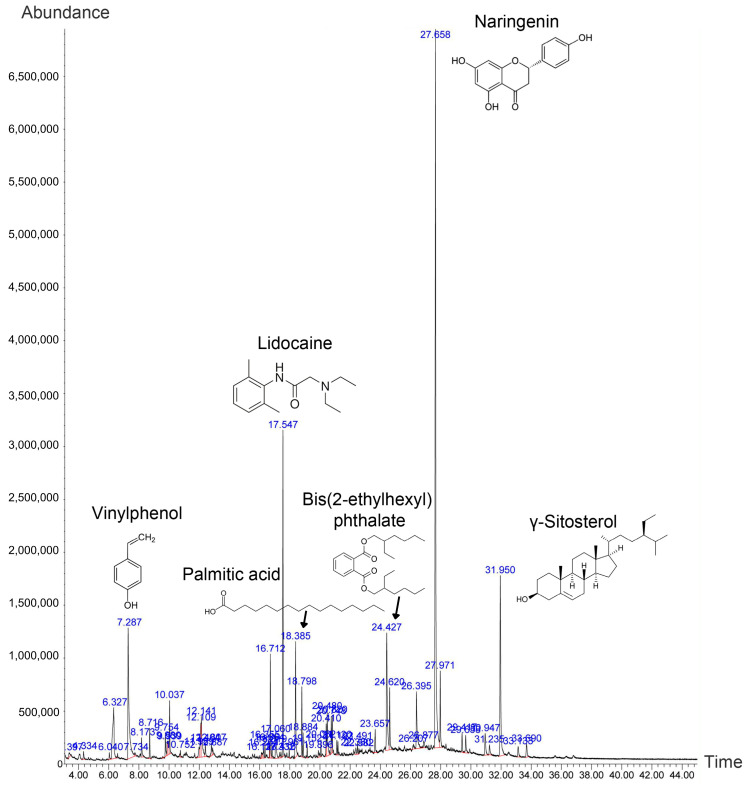
GC-MS chromatogram of the ethanolic husk extract from sacha inchi. The *Y*-axis indicates compound abundance, while the *X*-axis represents retention time. The six most abundant compounds, along with their names and chemical structures, are annotated.

Extraction (following 14 days of continuous soaking in 95% ethanol) from powdered *P. volubilis* husk (100 g) obtained a 0.65% yield (*w*/*w*). Phytochemical screening of the husk extract presented a total phenolic content of 41.49 ± 0.04 mg GAE/g and a total flavonoid content of 74.30 ± 0.23 mg QE/g. The gas chromatography–mass spectrometry (GC-MS) chromatogram showed approximately 43 peaks (Figure 1), while the analysis confirmed the presence of 43 distinct compounds (Table S1). Naringenin was the most abundant compound detected, followed by γ-sitosterol, lidocaine, 4-vinylphenol, benzoic acid, and palmitic acid (at 27.57, 7.74, 7.41, 7.00, 4.07, and 3.62%, respectively). Other compounds were found in relatively low quantities.

Liquid chromatography–mass spectrometry (LC-MS) analysis was additionally performed on the husk ethanol extract to identify compounds missed by GC-MS analysis. A total of 55 compounds were identified in positive mode (Table S2) and 38 compounds in negative mode (Table S3). Of these, twenty-three compounds were phenolic, including four phenolic acids, five flavonoids, and fourteen other phenyls, while the remaining compounds comprised mainly fatty acids and alkaloids, among others.

### 2.2. Cytotoxicity of the Husk Extract Against Cell Lines

**Figure 2:**
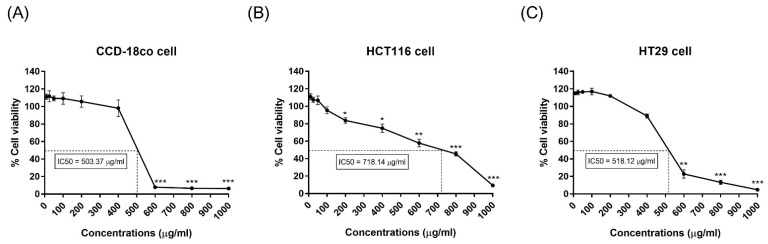
Cytotoxic potential of the husk extract against colon cell lines. Graphs demonstrate the results of MTT assays following exposure to various concentrations of the husk extract (10–1000 µg/mL) for 24 h. The treatments included (**A**) normal colon cells (CCD-18co) and the colon cancer cells (**B**) HCT116 and (**C**) HT29. Data were analyzed using a paired *t*-test and are presented as the mean ± SD. *p*-values are indicated by asterisks: * *p* < 0.05; ** *p* < 0.01; *** *p* < 0.001.

MTT assays were performed to evaluate the effect of the husk extract on cell viability. Normal colon cells (CCD-18co) and colon cancer cells (HCT116 and HT29) were separately treated with the husk extract at various concentrations (10, 25, 50, 100, 200, 400, 600, 800, and 1000 µg/mL) for 24 h. Cells treated with 1% DMSO were used as sham controls. CCD-18co cell viability was significantly reduced following treatment with 600 µg/mL of the husk extract or more (Figure 2A). HCT116 cells showed a reduced cell viability following treatment with 50 µg/mL of the husk extract; however, it was only significant from 200 µg/mL and higher (Figure 2B). HT29 cell viability decreased at 200 µg/mL of husk extract and was significant from 400 µg/mL and higher (Figure 2C). Furthermore, the half-maximal inhibitory concentration (IC_50_) of the husk extract for CCD-18co, HCT116, and HT29 cells was 503.37, 718.14, and 518.12 µg/mL, respectively. The highest concentration of husk extract that demonstrated continued cell viability (95–105%) was selected for further experiments.

### 2.3. The Husk Extract Inhibits the Migration and Invasion of Colon Cancer Cells

**Figure 3:**
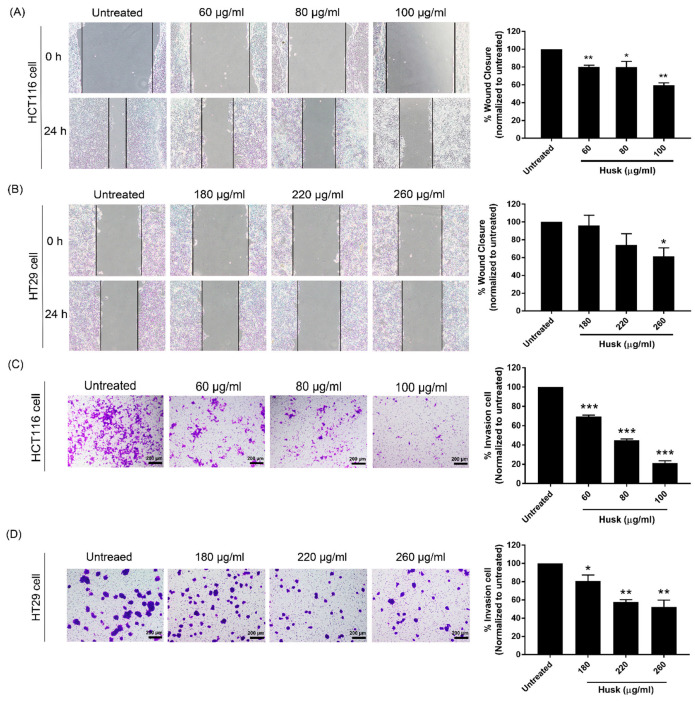
Analysis of the husk extract’s effect on the migration and invasion of HCT116 and HT29 cells. Representative images showing the morphology and quantification of colon cancer cells (**A**) HCT116 and (**B**) HT29 at 0 and 24 h post-treatment using various concentrations of the husk extract. Representative images showing cell invasion post-treatment for (**C**) HCT116 cells and (**D**) HT29 cells treated with various concentrations of the husk extract. Scale bars in (**C**,**D**) represent 200 µm. Data were compared using a paired *t*-test and presented as the mean ± SD. Asterisks indicate the *p*-values: * *p* < 0.05; ** *p* < 0.01; *** *p* < 0.001.

The effects of the husk extract on colon cancer cell migration were investigated using wound scratch assays. Treatment with the husk extract significantly decreased wound closure in HCT116 and HT29 cells at 60 and 260 µg/mL, respectively, compared to the untreated group (Figure 3A,B). Higher extract concentrations appeared to further induce wound closure compared to the lowest concentration that elicited a statistically significant effect. Assessment of cell invasion was also performed using a Transwell assay. A significant decrease in cell invasion was observed in the husk extract-treated groups at all tested concentrations, compared to the untreated group (Figure 3C,D). The percentage of cell invasion in HCT116 and HT29 cells treated with the husk extract occurred in a dose-dependent manner.

### 2.4. Effect of the Husk Extract on the Colony Formation of Colon Cancer Cell Growth

**Figure 4:**
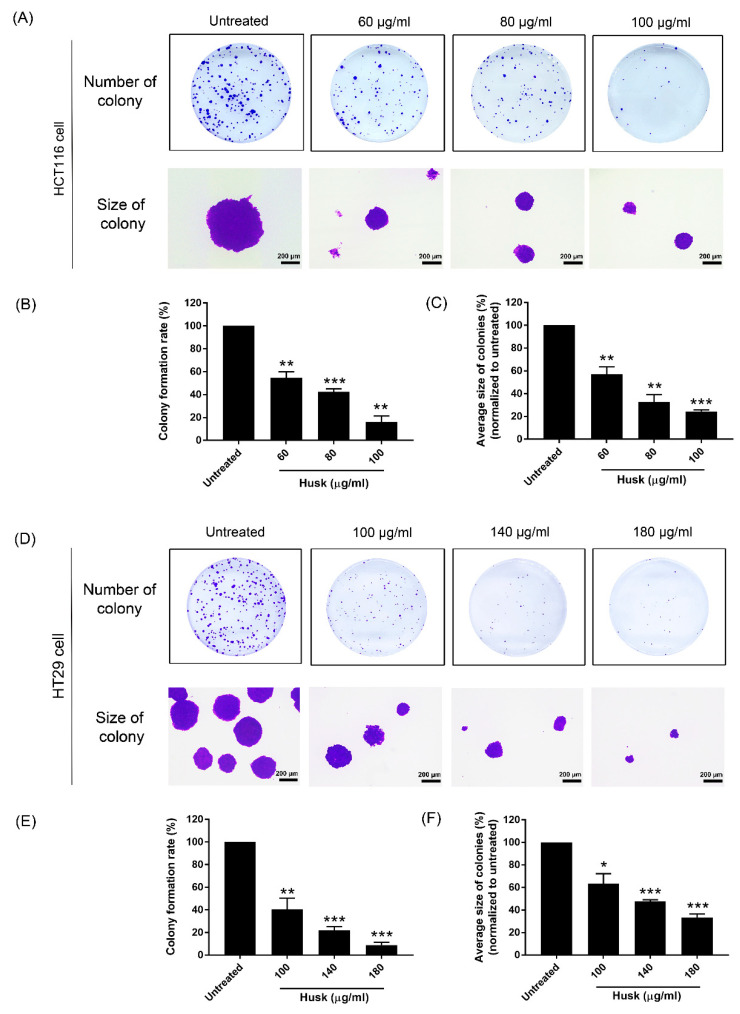
The husk extract’s effect on cancer cell colony formation. (**A**) The number and size of the colonies for HCT116 cells treated with the husk extract at concentrations of 60, 80, and 100 µg/mL. (**B**,**C**) The percentages of colony formation and the average size of the colonies for HCT116 cells, respectively. (**D**) The number and size of the colonies for HT29 cells treated with the husk extract at concentrations of 100, 140, and 180 µg/mL. (**E**,**F**) The percentages of colony formation and the average size of the colonies for HT29 cells, respectively. Data in histograms are presented as the mean ± SD, with asterisks indicating statistical differences as follows: * *p* < 0.05; ** *p* < 0.01; *** *p* < 0.001.

A colony formation assay was used to study the effect of the husk extract on cancer cell growth and colony formation. The husk extract significantly reduced the number and average size of colonies at 60 µg/mL for HCT116 cells (Figure 4A–C) and 180 µg/mL for HT29 cells (Figure 4D–F) when compared to the untreated group.

### 2.5. Effect of Husk Extract Treatment on the Gene Expression of Colon Cancer Cells

**Figure 5:**
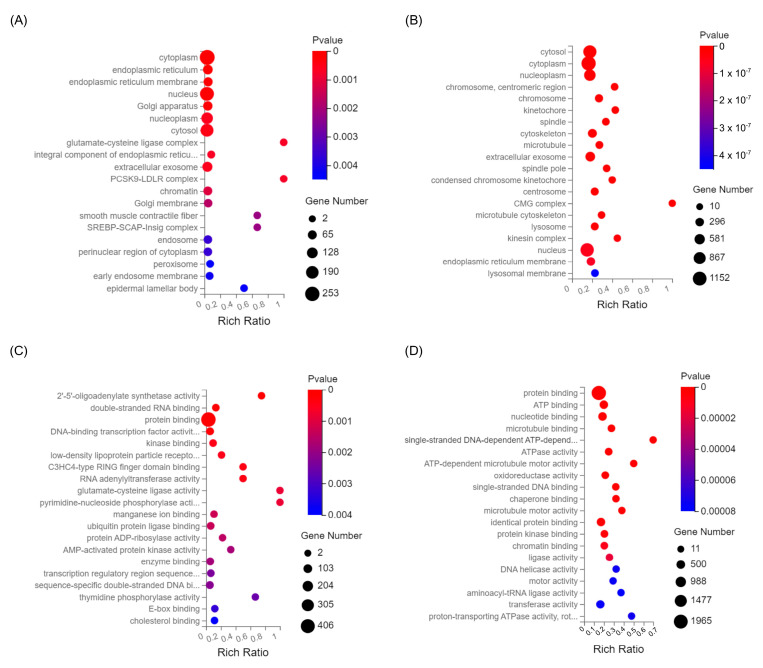
GO classification for all significantly differentially expressed genes in HCT116 and HT29 cell lines following husk extract treatment. Classification for GO cellular component enrichment in (**A**) HCT116 and (**B**) HT29 cells. Classification for GO molecular function enrichment for (**C**) HCT116 and (**D**) HT29 cells. In the bubble diagrams, the circle size represents the number of enriched genes, and the color represents the significance of enrichment.

Differential RNAseq analysis was used to investigate the effect of the husk extract on colon cancer cells at the molecular level (Table 1). Of the significantly upregulated differentially expressed genes (DEGs), 47.10% (3725 genes) were shared between the HCT116 and HT29 cell lines, while 46.88% (3822 genes) of the significantly downregulated DEGs were shared. Subsequent KEGG pathway analysis of all DEGs demonstrated that the majority were involved in cellular processes, environmental information processing, genetic information processing, human diseases, metabolism, and organismal systems (Figure S1). The human disease category contained the highest number of DEGs, within which the viral, bacterial, and cancer groups were most abundant. Signal transduction, within the environmental information processing category, had the highest number of representative DEGs. Gene Ontology (GO) analysis of all DEGs within the cellular component for both HCT116 and HT29 cells revealed an enrichment of genes that encoded components associated with the cytoplasm and nucleus (Figure 5A,B). For molecular function, both cell lines displayed a large enrichment of genes associated with protein binding (Figure 5C,D). Other categories of interest included kinase binding, manganese ion binding, enzyme binding, and E-box binding for the HCT116 cells (Figure 5C), while ATP binding, ATPase binding, chaperone binding, and chromatin binding were most enriched for HT29 cells (Figure 5D).

**Figure 6:**
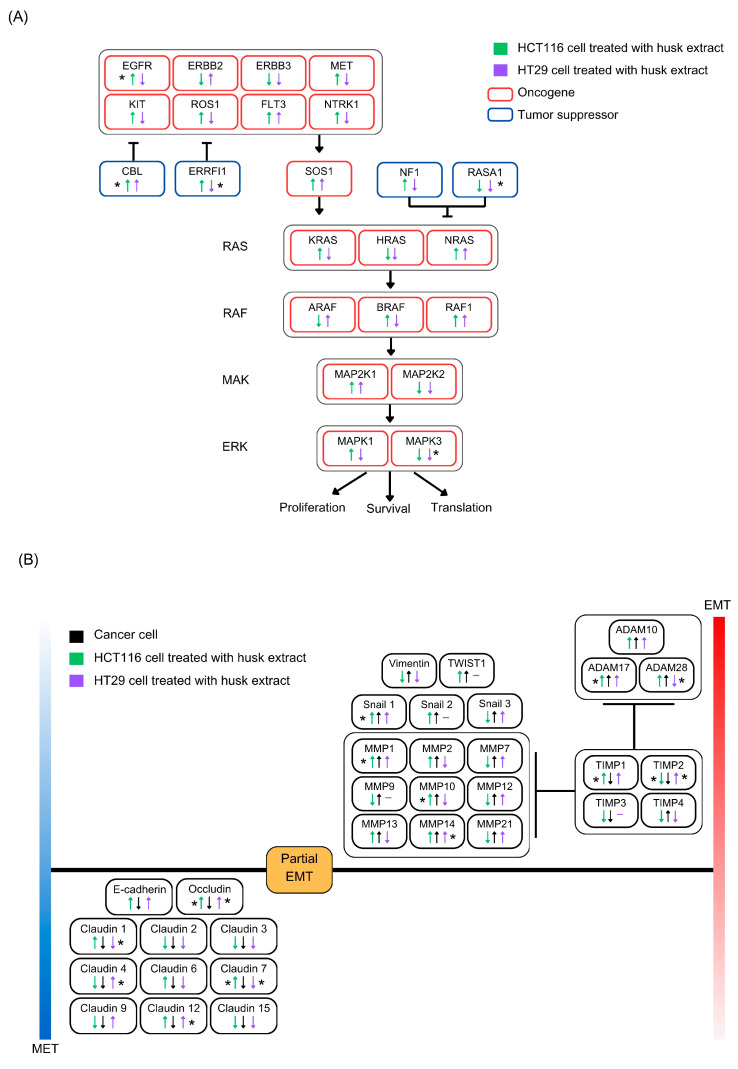
Comparative expression of the genes involved in upstream and downstream cancer pathways following husk extract treatment on cancer cell lines. Expression of genes in the (**A**) upstream EGFR pathway and the (**B**) downstream EMT pathways for HCT116 and HT29 cells treated with the husk extract. The blue and red bars represent the level and intensity of gene expression, respectively. EMT and MET are non-binary, reversible processes that emphasize the plasticity of cells to transition between these two states. Data were compared using a paired *t*-test. * *p* < 0.05.

Comparative expression of the genes involved in upstream (epidermal growth factor receptor, EGFR) and downstream (epithelial–mesenchymal transition (EMT) and mesenchymal–epithelial transition [20]) cancer pathways was targeted for investigation. For the upstream pathway, EGFR and CBL genes were significantly upregulated in HCT116 cells, while ERRFI1, RASA1, and MAPK3 genes were significantly downregulated in HT29 cells when compared to the untreated group (Figure 6A and Figure S2). Additionally, we observed an oppositional expression of significant genes such as EGFR and ERRFI1 between HCT116 and HT29 cells. For the downstream pathway, significant upregulation and downregulation of several genes, including SNAI1 and MMPs, in both cancer cell lines were identified, compared to the untreated group (Figure 6B and Figure S3).

### 2.6. Effect of Husk Extract Treatment on Colorectal Cancer Cell Proteomes

**Figure 7:**
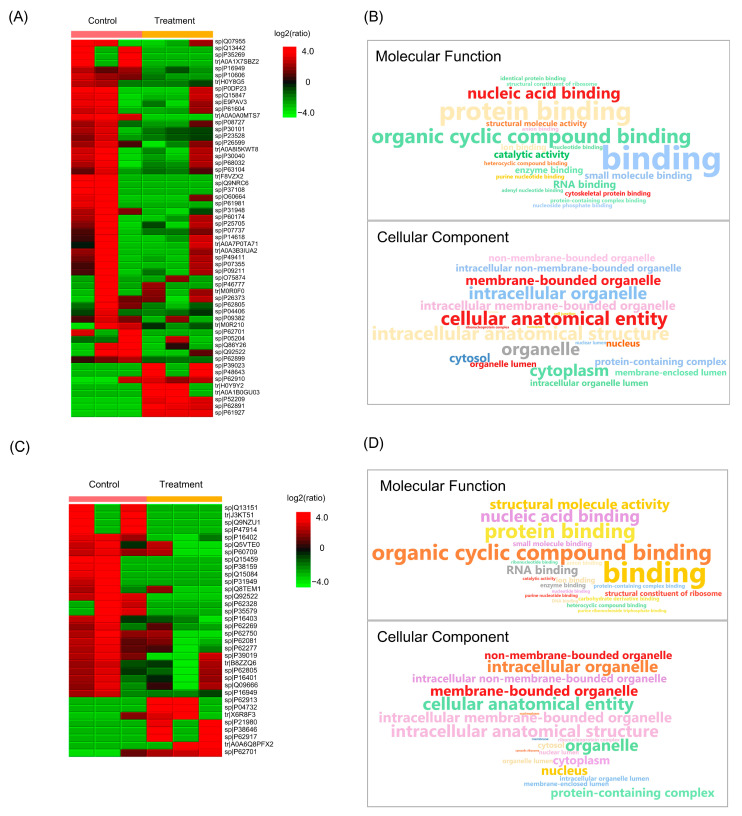
Proteomic analysis of HCT116 and HT29 cells treated with 100 µg/mL and 250 µg/mL of the husk extract, respectively. (**A**,**C**) Heat maps showing the relative protein abundance in HCT116 and HT29 cells following treatment, compared to the control (untreated cells). Each group contained three biological replicates. (**B**,**D**) Word clouds showing the cellular components and molecular functions for identified proteins in HCT116 and HT29 cells, respectively.

Semi-quantitative proteomic analysis of HCT116 and HT29 cells following husk extract treatment identified 52 and 30 differentially abundant proteins, respectively, and four of the same proteins were found in both cell lines. In both cell types, the majority of proteins were relatively abundant in the control groups and associated with similar molecular functions and cellular components (Figure 7 and Table S6). For example, GO terms relevant to binding, organic cyclic compound binding, protein binding, and nucleic acid binding were enriched. Regarding cellular components, cellular anatomical entities, intracellular organelles, and membrane-bounded organelles were enriched in both cell lines.

**Figure 8:**
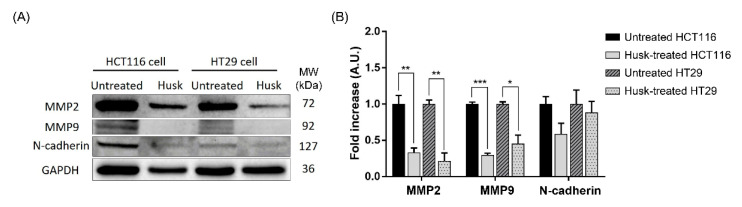
Western blot showing the effects of the husk extract on the abundance of proteins associated with the downstream EMT pathway. (**A**) Representative Western blot image showing localization of MMP2, MMP9, N-cadherin, and GAPDH proteins in control (untreated cells) and treatment (husk extract) groups. The molecular weight (MW) of each protein is provided. (**B**) Quantitative analysis of protein levels following Western blot analysis. Data were compared using a paired *t*-test and are presented as the mean ± SD. *p*-values are indicated by asterisks: * *p* < 0.05; ** *p* < 0.01; *** *p* < 0.001.

A Western blot was performed to investigate changes in the proteins involved in the downstream EMT pathway, including MMP2, MMP9, and N-cadherin. In both HCT116 and HT29 cells, MMP2 and MMP9 protein abundance decreased significantly following husk extract treatment (Figure 8 and Figure S4). Meanwhile, N-cadherin decreased following treatment, but was not significantly different from the untreated group.

## 3. Discussion

Sacha inchi husks are often considered low-value by-products of sacha inchi oil extraction and are mostly applied to fertilizer or animal feed. However, most studies have focused on the husk’s nutritional content, while the chemical properties potentially associated with medicinal applications remain largely unexplored. In this study, we reported the semi-purification of husk compounds that exhibit anticancer properties against colon cancer cells. We additionally provided insights into what molecular components change (i.e., gene expression and proteins) in colon cancer cells following husk extract treatment.

Studies focused on the nutritional content of husks, particularly the shell, have noted that they contain a total phenolic compound (TPC) level ranging from 3.24 to 129.9 mg GAE/g, depending on the extraction solvent used. The most effective extraction solvents are acetone-containing mixtures, such as methanol/acetone/water (80:19:1 and 40:40:10:1) at 60 °C [21]. In addition, a process where the husks were soaked in 50% aqueous ethanol for 24 h, followed by heating at 70 °C for 2 h, led to a much higher TPC content (129.9 mg GAE/g) compared to soaking with pure ethanol or water [22]. Other methods, like microwave-assisted extraction and hot water extraction, produced TPC levels of 41.97 mg GAE/g [23] and 74.8 mg GAE/g dry weight [24,25], respectively. Interestingly, heating the shell overnight at 50 °C had little impact on the TPC levels [25]. However, prior to the current study, only one study had looked explicitly at husk extracts, finding TPC levels of 3.24 and 5.04 mg GAE/g DW in the husk and shell, respectively, when using 20% (v/v) aqueous ethanol at 70 °C for 15 min [26]. In our study, husks extracted with 95% ethanol for 14 days showed a TPC level of 41.49 mg GAE/g extract and a total flavonoid content (TFC) of 74.30 mg QE/g extract. We speculate that this higher concentration was due to the extended soaking and incubation process, which enabled extra breakdown of the plant’s cell walls, making it easier to extract phenolic compounds when using a suitable solvent, temperature, and duration [27].

In earlier studies, phenolics from husk extracts were identified as flavonoids (e.g., quercetin, kaempferol, and isorhamnetin), phenolic acids (e.g., caffeic acid, gallic acid, 4-hydroxybenzoic acid, and p-coumaric acid), and palmitic acid [26,28]. Similarly, our findings showed a high concentration of phenolics and flavonoids, although GC-MS and LC-MS indicated higher amounts of naringenin and lidocaine, which had not been reported before. These differences could be due to variations in analytical methods, such as using GC-MS (instead of acidic methanol-based HPLC) or differences in the geographical location from which the plant samples were sourced. A previous study has shown that at high abundance, naringenin can inhibit colorectal cancer cell growth by repressing the PI3K/AKT/mTOR signaling pathway (Table 2) [29].

Our study is the first to explore the potential of sacha inchi husk extract in mitigating colon cancer cell growth, and in vitro cell assays confirmed the extract’s activity. In support of this, there was a significant reduction in HCT116 and HT29 cell viability and inhibition of colony formation, indicating strong antiproliferative effects. Additionally, the extract demonstrated an inhibitory effect on the migration and invasion of these cancer cell lines. Specifically, at concentrations exceeding 60 µg/mL on HCT116 cells and 180 µg/mL on HT29 cells, it effectively suppressed cell migration and invasion. This suggests that the extract may help prevent cancer cells from adhering to the epithelial surface at higher, yet biologically safe, concentrations. However, the results also indicated that different treatment concentrations are required for each cell line. These findings are consistent with previous research, where sacha inchi peptides showed potential in treating liver cancer [46].

Another significant finding in our study was the high level of naringenin identified in the extract, which could be the primary active compound against cancer cell lines; the pure compound has anticancer effects on various colorectal cancer cell lines, such as HCT116, SW620, SW1116, and SW480 [29,47]. At the cellular level, naringenin exerts anticancer effects through inhibiting the RAS/PI3K/AKT/mTOR pathway, inducing autophagy and apoptosis [48,49]. Another study found that naringenin works by inducing cell death (apoptosis), specifically through influencing the cell cycle, inhibiting RAS signaling, promoting autophagy (self-destruction of cancer cells), reversing drug resistance, and damaging the DNA of cancer cells [50]. It is possible that the effects observed on colon cancer cells are due to the sacha inchi husk-derived naringenin; however, further targeted analysis is required to confirm the exact active components.

Our transcriptome results indicated that the husk extract could potentially inhibit cell proliferation, survival, and translation in HCT116 and HT29 cells through the upstream EGFR pathway. In particular, compared to the untreated control group, the extract upregulated CBL tumor suppressor genes in HCT116 cells and downregulated MAPK3 oncogenes in HT29 cells. The upstream PTK/RAS pathway has been implicated in the regulation of several biological processes, including cell proliferation, differentiation, angiogenesis, apoptosis, and survival [51]. As mentioned, the EGFR/MAPK signaling pathway is involved in the oncogenic process and thus plays a key role in tumor growth and the progression of colon cancer [52,53]. Furthermore, aberrant expression of this pathway has been reported as a potential therapeutic target for colon cancer [54,55]. Alterations in CBL and MAPK3 reflect a balance of signaling pathways involved in cancer cell growth and survival, particularly the EGFR/MAPK pathway, which plays a key role in stimulating cell proliferation, evading apoptosis, and metastasis. The downregulation of MAPK3 may reflect inhibition of signaling through the ERK cascade, while the upregulation of CBL, an E3 ubiquitin ligase that regulates EGFR degradation, may be a mechanism to reduce excessive EGFR activation [56,57]. Although the EGFR pathway is considered unresponsive to treatment in patients with KRAS mutations, recent research in 2025 found that EGFR still plays an active role in colon cancer cells with KRASG12D mutations. Inhibiting EGFR can significantly alter cancer cell metabolism, such as decreasing glycolysis and increasing glutamine utilization, and induce the expression of genes associated with patient survival [56]. This study suggests that EGFR blockade may still be beneficial in patients with KRAS mutations if used in combination therapies such as pan-KRAS inhibitors or ligand blockers [58]. Therefore, the CBL and MAPK3 changes observed in this study may reflect a rebalancing of EGFR signaling that can lead to inhibition of cancer cell growth, even in the presence of KRAS mutations. This information is crucial for the development of future targeted therapeutic strategies [59].

Interestingly, lidocaine was found to be the second most abundant compound in the husk extract. Previous research has shown that lidocaine has antiproliferative effects on colorectal cancer cells (SW480 and HCT116), causing cell death by suppressing the EGFR through the upregulation of miR-520a-3p, which targets EGFR [37]. Lidocaine also inhibited the growth of colon cancer cell lines HCT116 and RKO by regulating critical proteins involved in apoptosis, such as caspase-8, p53, survivin, HSP-27, and HSP-60 [60]. However, another study found that lidocaine caused cell cycle arrest in HT-29 and SW480 colon cancer cells and did not significantly affect cell proliferation or induce cell death in these lines [61]. Our findings are consistent with those of previous studies. The husk extract inhibited the growth of colon cancer cell lines HCT116 and HT29 through the EGFR pathway, as indicated by the gene expression data.

The comparative RNAseq and proteomics analysis supported the in vitro assays, showing molecular-level changes within downstream targets, with overall downregulation following treatment. The downstream EMT pathways were the primary pathways with the most significant cell inhibition genes (Table S4). Furthermore, they showed the same direction, with most proteins exhibiting protein-binding properties and being associated with EMT-related proteins. The husk extract significantly decreased the levels of MMP9 and increased the tissue inhibitors of metalloproteinase-1 (TIMP-1) in both cancer cell lines. Additionally, in HT29 cells, MMP2 levels dropped while TIMP-2 levels rose. Cancer cell migration and invasion, which lead to metastasis, are driven by various signaling pathways [62,63] that require enzymes like serine proteinases and MMPs, which break down the ECM. Specifically, MMP2 and MMP9 are frequently overexpressed in aggressive tumors and play a key role in degrading the ECM, particularly the basal membrane, thus contributing to cancer metastasis [64,65]. Meanwhile, TIMPs are natural regulators of MMP activity [20]. Hence, maintaining a balance between MMPs and TIMPs is critical, as a disruption in this balance can lead to ECM degradation and accelerate the spread of cancer cells [66]. Another critical process involved in metastasis is EMT, in which cancer cells lose their ability to adhere to each other, enhancing their capacity to invade surrounding tissues and distant organs [67,68]. A decrease in N-cadherin, a marker of epithelial cells, is a hallmark of EMT [69,70].

To support the gene expression results, we performed protein expression analysis of the key proteins associated with cancer progression, including MMP2 and MMP9, which are involved in ECM degradation, and N-cadherin, which plays an important role in cancer cell invasion and metastasis [71,72]. We found that both MMP2 and MMP9 were significantly reduced in both colon cancer cell lines, suggesting that changes at the protein level were more pronounced than at the gene level. N-cadherin was also decreased in the husk-treated groups, although no statistically significant difference was observed when compared to the controls. These findings align with previous research, such as a study by Chang et al. [47], which showed that naringenin effectively reduced the migration and invasion of colon cancer cells by lowering the expression of MMPs and N-cadherin, both of which are critical for ECM and EMT regulation. Our findings are also consistent with a study by Li et al. [73], which demonstrated that glabridin, a flavonoid and one of the main active compounds in the flowering plant *Glycyrrhiza glabra* (licorice), inhibits the proliferation, migration, and invasion of SW480 and SW620 colorectal cancer cells, while also inducing apoptosis.

Although transcriptomic and proteomic analyses revealed changes in the expression of genes and proteins related to the EGFR and EMT/MET pathways in the extract, the current data cannot clearly demonstrate a causal relationship between these changes and the observed phenotypes (e.g., reduced cell growth and cell motility) due to the lack of functional validation experiments, such as pathway inhibition, knockdown, or overexpression of target genes, or rescue assays. Therefore, the link between alterations in the EGFR and EMT/MET pathways and the anticancer effects of the extracts remains correlative and cannot be confirmed as the primary mechanism of action. Other pathways also exhibited alterations, which may play a contributing role in the development of these phenotypes. The authors recognize this limitation and plan to conduct further experiments in the future to confirm the role of the relevant pathways, particularly the EGFR and EMT/MET pathways, through the use of pathway inhibitors, siRNA-mediated knockdown, or rescue assays to generate more definitive mechanistic evidence.

## 4. Materials and Methods

**Figure 9:**
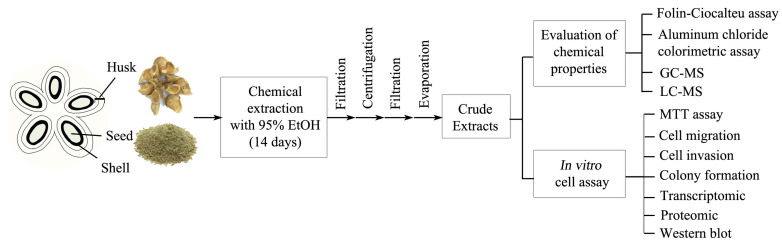
The overall workflow for this research. Sacha inchi husk compounds were extracted and analyzed through chemical analysis and in vitro effects on colon cancer cells.

The overall workflow for this research investigation is shown in Figure 9.

### 4.1. Plant Materials and Extraction

The husk of sacha inchi was obtained from TAI.C.M.S. STANDARD INDUSTRIAL CO., LTD., Chiang Rai Province, Thailand. The husk was extracted with 95% ethanol for 14 days at 60 °C in a water bath, with shaking every 10 min, to produce an ethanol fraction (husk/ethanol ratio, 100 g:1.5 L). Then, it was filtered with a thin white cloth, and the filtered waste was immersed in 95% ethanol and filtered with a thin white cloth again. This was repeated until the filtered water was clear. After this, the extracts were centrifuged at 12,000× *g* and 25 °C for 10 min. Then, the extracts were filtered with a 6 µm filter (Whatman^®^ qualitative filter paper, Grade 3, Merck, Darmstadt, Germany) using a vacuum flask and separated from the ethanol using a rotary evaporator at 56 °C. The crude extract was dissolved in 100% DMSO as a 1 mg/mL stock solution and stored at −20 °C.

### 4.2. Phytochemical Evaluation

#### 4.2.1. Determination of Total Phenols by Folin–Ciocalteu Reagent Method

Gallic acid was used as the standard polyphenolic compound (4.7–300 µg/mL) for plotting the calibration curve. The gallic acid and husk extract samples were prepared in dimethylsulfoxide (DMSO), following which 20 µL was added to a well and mixed with 100 µL of Folin–Ciocalteu reagent (10% *w*/*v*). The mixture was incubated in a dark room at room temperature for 5 min and then neutralized with 80 µL of sodium carbonate solution (25%, *w*/*v*). After a 20 min incubation, the reaction mixture was measured at 760 nm with a microplate spectrophotometer (VersaMax microplate reader, Marshall Scientific, Hampton, VA, USA) against distilled water as a blank. The phenolic content is expressed as micrograms of gallic acid equivalent (GAE/mg) of dry extract.

#### 4.2.2. Determination of Total Flavonoid Content by Aluminum Chloride Colorimetric Method

Quercetin was used as the standard flavonoid content (4.7–300 µg/mL). A total of 20 µL of the quercetin and husk extract samples was added to a well and mixed with 180 µL of aluminum chloride (2% *w*/*v*). The test well was incubated for 10 min at room temperature. The absorbance was measured at 415 nm using a microplate spectrophotometer (VersaMax microplate reader) against distilled water as a blank. The flavonoid content is expressed as micrograms of quercetin equivalent (QE/mg) of dry extract.

### 4.3. Gas Chromatography–Mass Spectrometry (GC-MS) Analysis

An aliquot of the dried husk of sacha inchi ethanol extract (15.40 mg) was diluted in 1.5 mL of ethanol. The sample was injected into a GC-MS system consisting of gas chromatography (Agilent 7890B, Agilent, CA, USA) and a mass spectrometer detector (Agilent 5977B, Agilent, CA, USA). A ZB-5MS UI capillary column (30 m × 0.25 mm, 0.25 µm film thickness, Agilent, CA, USA) was used. The inlet was set to 280 °C and split mode with a ratio of 10:1. Helium gas was used as the carrier gas and was adjusted to a column velocity flow of 1 mL/min. The temperature program was as follows: starting at an initial temperature of 80 °C and then ramped first to 100 °C at a rate of 5 °C/min and finally to 300 °C at a rate of 8 °C/min, then held for 16 min, with a total run time of 45 min. The result was calculated by comparing its average peak area to the total area. Identification of components was achieved based on their retention using the database of the National Institute of Standards and Technology (NIST).

### 4.4. Liquid Chromatography–Mass Spectrometry (LC-MS) Analysis

Phytochemical profiling of the husk ethanol extract and fractions was determined using UltiMate^TM^ NCS-3500RS Binary Rapid Separation Nano/Capillary Pumps (Thermo Fisher Scientific, Waltham, MA, USA). The mobile phases were prepared with solvent A, deionized water with 0.1% formic acid, and solvent B, acetonitrile with 0.1% formic acid. Gradient elution was performed at a 2.5 mL/min flow rate at room temperature and a pressure limit of 620 bar. The elution gradient program started with 5% of solvent B for 1 min, before gradually increasing this to 95% of solvent B for 20 min, which was maintained for 4 min, before being reducing to 5% solvent B and maintained for another 5 min.

Mass spectrometric ionization was performed in negative ion mode. The run duration was 30 min, consisting of 545 cycles of 3.3029 s each. The range of mass detected was from 100 to 2000 Da for MS1 and from 50 to 2000 Da for MS2. The MS1 acquisition conditions included nebulizer gas (GS1: nitrogen), drying gas (GS2: nitrogen), and curtain gas (CUR); their flow rates were 50, 60, and 30 psi, respectively. The temperature was 500 °C, and the ion spray voltage was 4500 V. For MS2 acquisition, a decluttering potential of 80 V, a collision energy (CE) of 40 V, and a collision energy spread (CES) of 10 V were applied, respectively. Positive ion mode analysis was performed using the same parameters, but with an ion spray voltage of 5500 V.

### 4.5. Cell Cultures

The normal colon (CCD-18Co) and two colon cancer (HCT116 and HT29) cell lines were purchased from the American Type Culture Collection (ATCC). Briefly, CCD-18Co was grown in DMEM with 1 g/L of D-glucose and L-glutamine, 110 mg/L of sodium pyruvate, 10 U/mL of penicillin G, 10 µg/mL of streptomycin, and 10% fetal bovine serum (cell culture medium). HCT116 and HT29 cells were grown in McCoy’s 5A medium with L-glutamine, without sodium bicarbonate, penicillin G (10 U/mL), streptomycin (10 µg/mL), and 10% fetal bovine serum (cell culture medium). All cell lines were cultured at 37 °C in a humidified incubator with a 5% CO_2_ atmosphere. The culture medium was changed every 2–3 days. Cells at the logarithmic stage were harvested before performing each experiment.

### 4.6. MTT Assay

Cell (CCD-18Co, HCT116, and HT29) viability was measured using an MTT assay. The cells were seeded into 96-well plates at 1 × 10^4^ cells per well, containing 100 µL of the respective culture medium, and allowed to grow for 24 h at 37 °C. After this time, the cells were treated with various concentrations of the extract (10–1000 µg/mL) or 1% DMSO as a negative control for 24 h. After treatment, MTT was added to each well and incubated for 2 h at 37 °C. Finally, the MTT was removed, and 100 µL/well DMSO was added. Absorbance was determined using a microplate spectrophotometer (VersaMax microplate reader) at wavelengths of 570 and 690 nm. The percentage of viable cells was calculated after normalization with the negative control, which was considered 100% cell viability and calculated using the following equation:

$$\%\;Cell\;viability=\left(\frac{{Absorbance}_{\left(sample\right)}}{{Absorbance}_{\left(negative\;control\right)}}\right)\times 100$$

### 4.7. Cell Migration by Wound Scratch Assay

The cell migratory ability of HCT116 and HT29 cells was determined using a wound scratch assay. Cells were seeded into 6-well plates at 1 × 10^6^ cells per well, containing 2 mL of the respective culture medium, and were allowed to grow for 24 h at 37 °C. After this time, the monolayer cells were scratched using sterile pipette tips. The medium was discarded, and the cells were treated for 24 h with various concentrations of the extract. Five concentrations were selected based on the percentage of cell viability, ranging from 105% to 95%, with untreated cells serving as the negative control. The analysis involved removing the plate from the incubator and placing it under an inverted microscope to take a snapshot and check for wound closure. Experiments were carried out in triplicate, and four fields of each point were recorded. ImageJ software (version 6.1) was used to measure the scratched area. The cell migratory ability during wound healing was assessed using the following formula:

$$\%\;Wound\;Closure=\left[\frac{A_{t\;=\;0\;h}-A_{t\;=\;\Delta h}}{A_{t\;=\;0\;h}}\right]\times 100$$

A*_t_*_= 0_ *_h_* is the area of the wound measured immediately after scratching (*t* = 0 *h*).

A*_t_*_= Δ_*_h_* is the area of the wound measured h hours after the scratch was performed.

### 4.8. Cell Invasion by Transwell Assay

This method used a Corning Matrigel Basement Membrane Matrix (Corning 356234, Corning, NY, USA). The Matrigel matrix was diluted in serum-free medium to a final concentration of 20 µg in the center of each Transwell^®^ insert (8 µm PET membrane, Corning 3464, Corning, NY, USA). The plate was incubated at 37 °C for 1 h to allow the Matrigel matrix to form a gel. After that, HCT116 and HT29 cells were seeded at 250 µL into the upper chamber of each Transwell insert. The final cell density was 5 × 10^4^ cells per well using the treatment extract in serum-free medium (supplemented without fetal bovine serum). At the same time, 800 µL of medium (supplemented with 20% fetal bovine serum) was used as a chemoattractant added to the lower chambers after 24 h of culture in a humidified incubator at 37 °C with 5% CO_2_. The Transwell inserts were washed twice with phosphate-buffered saline (PBS). The cells inside the Transwell inserts were gently removed using moistened cotton swabs. After this, the cells on the lower surface of the membrane were fixed with the inserts using absolute methanol for 5 min and then stained with 0.1% crystal violet for 3 min. The Transwell inserts were washed twice with PBS to remove unbound crystal violet. Invaded cells that migrated through the pores to the lower surface of the filter were observed and counted under a microscope. The values were calculated by averaging the total number of cells from three filters. Cell invasion ability was assessed using the following formula:

$$\%\;Invasion=\left(\frac{{Number\;of\;cell\;invade}_{\left(sample\right)}}{{Number\;of\;cell\;invade}_{\left(control\right)}}\right)\times 100$$

### 4.9. Colony Formation

Cultured HCT116 and HT29 cells in the logarithmic phase were seeded at a density of 1 × 10^3^ cells per well into 6-well plates containing 2 mL of the respective culture medium and allowed to grow for 24 h at 37 °C. After this time, the medium was discarded, and the cells were treated for 24 h with various concentrations of the extract. After incubation at 37 °C for 10 days, when macroscopic cell colonies appeared on the bottom of the plate, the cells were washed twice with PBS and fixed with absolute methanol for 5 min. Then, the cell colonies were stained with 0.1% crystal violet for 3 min. The cells were washed twice with PBS to remove unbound crystal violet, and the number of cell colonies was visually measured using a microscope (clusters containing 50 cells were counted as a single colony). The analysis of colony formation efficiency was defined as the number of cell colonies and the size of the colonies. ImageJ software determined the colony size using the following formula:

$$\%\;Colony\;formation\;rate=\;\left(\frac{{Number\;of\;colony}_{\left(sample\right)}}{{Number\;of\;colony}_{\left(control\right)}}\right)\;\times 100$$

$$\%\;Average\;size\;of\;colonies=\left(\frac{{Average\;size\;of\;colonies}_{\left(sample\right)}}{{Average\;size\;of\;colonies}_{\left(control\right)}}\right)\times 100$$

### 4.10. Transcriptome

The cells were cultured and seeded into 6-well plates containing 2 mL of McCoy’s 5A (Modified) Medium (Gibco™, Thermo Fisher Scientific, MA, USA) at a density of 1 × 10^6^ cells per well. The cells were allowed to grow for 24 h at 37 °C and 5% CO_2_. After this, the medium was discarded, and the cells were treated with the husk extract at 100 µg/mL for the HCT116 cells and 250 µg/mL for the HT29 cells at 37 °C for 24 h. Once the incubation was completed, the treated cells were scraped and centrifuged at 1000× *g* for 5 min at room temperature (RT). Next, cell pellets were washed with PBS once, and the pellet was kept for RNA extraction.

RNA isolation was carried out using TRIzol^TM^ Reagent (Thermo Fisher Scientific, #15596026, MA, USA). The cell pellets were homogenized and 0.75 mL of TRIzol^TM^ Reagent was added. After this, 0.2 mL of chloroform was added for lysis, then thoroughly mixed by shaking. The samples were incubated for 2 min and centrifuged for 15 min at 12,000× *g* and 4 °C. Then, the aqueous phase containing the RNA was transferred to a new tube. In the next step, the RNA was precipitated with 0.5 mL of isopropanol, incubated for 10 min at 4 °C, and centrifuged for 10 min at 12,000× *g* at 4 °C. The total RNA precipitate formed a white gel-like pellet at the bottom of the tube. The supernatant was discarded, and the RNA was washed with 1 mL of 75% ethanol. The sample was vortexed briefly, then centrifuged for 5 min at 7500× *g* at 4 °C, and the supernatant was discarded. The RNA pellet was air-dried for 10 min and then solubilized in 50 µL of RNase-free water containing 0.1 mM EDTA. Finally, the RNA was incubated in a water bath or heat block at 55 °C for 10 min. Downstream applications were then implemented, or the RNA was stored at −80 °C.

RNA sequencing (RNAseq) was then carried out via a commercially available service (BGI Hong Kong Tech Solution NGS Lab, Tai Po, Hong Kong). After total RNA was fragmented into short fragments, mRNA was enriched using oligo (dT) magnetic beads, followed by cDNA synthesis. Double-stranded cDNA was purified and enriched by PCR amplification, after which the library products were sequenced using BGIseq-500. Data were available in the NCBI’s Sequence Read Archive (SRA) under identifier PRJNA1236814. KEGG pathway and GO bioinformatics analyses were performed using the BGI by the Dr. TOM approach, an in-house customized data mining system. The database used for mapping and annotation was *Homo sapiens* by NCBI and Reference Genome version GCF_000001405.39_GRCh38.p13. The altered (upregulated or downregulated) expression of genes is expressed as log2FC, representing log-transformed fold change by FPKM level. Genes associated with upstream (PTK/RAS pathway) and downstream (EMT pathway) signaling, which have been well described in terms of migration and invasion of cancer cells [74], were targeted for gene expression investigation.

### 4.11. Sample Preparation and Proteomic Analysis by Quadrupole Time-of-Flight (QTOF) Mass Spectrometry

The cells were seeded in 6-well plates containing 2 mL of complete McCoy’s 5A medium at a density of 1 × 10^6^ cells per well and allowed to grow for 24 h at 37 °C and 5% CO_2_. After this time, the medium was discarded, and cells were treated for 24 h with the husk extract. After incubation at 37 °C for 24 h, the cells were scraped and centrifuged at 1000× *g* for 5 min at RT. Next, the cell pellets were washed with PBS and lysed with 600 µL of lysis buffer (8 M urea, 0.8 M NH_4_HCO_3_, pH 8.0) supplemented with phenylmethylsulfonyl fluoride (PMSF). The samples were then sonicated for 2 min on ice (40% amplitude, 10 s off/on cycle) and then centrifuged at 12,000× *g* and 4 °C for 15 min. The supernatants were collected, and the protein concentration in the cell lysates was measured using a Pierce BCA protein assay (Thermo Fisher Scientific^TM^, MA, USA). Then, 100 µg of proteins was reduced with 5 µL of 100 mM dithiothreitol (DTT) for 1 h at 37 °C and subsequently alkylated with 20 µL of 100 mM alkylating reagent (IAA) for 1 h at RT in the dark, followed by incubation with the addition of 20 µL of 200 mM DTT in 6 M urea at RT for 60 min. The urea concentration was reduced by diluting the reaction mixture with 775 µL of MilliQ water. Then, the proteins were digested with sequencing-grade modified trypsin (Promega) at a 1:50 enzyme-to-substrate ratio. After overnight (~16 h) digestion at 37 °C with shaking at 20 rpm in a shaker incubator, the digested samples were acidified with 10% formic acid (FA) to a pH < 3. Tryptic peptides were buffer-exchanged using Sep-Pak C18 cartridges (Waters, Milford, MA, USA) following the manufacturer’s procedure and dried using a Speed Vac instrument. Lyophilized tryptic peptides were dissolved in 50 µL of 0.1% FA.

The samples were loaded into an Exion LC liquid chromatography system (AB SCIEX, Concord, On L4k 4v8, Canada) and programmed to a specific sequence. The selected samples were injected into a C18 peptide column before programmed quantities of water and acetonitrile solvent were pumped through the column over a 52 min time gradient, separating the sample into its chemical components. The standard operations utilized a two-component solvent comprising LC-MS-grade 0.1% formic acid (solvent A) and acetonitrile and 0.1% formic acid (solvent B) as follows: we started with 100% solvent A for the first 5 min, which was reduced to 60% solvent A for 40 min and then 5% for 5 min, and then this was maintained for 2 min before returning to 100% solvent A to reset the column. For the peptide samples, 15 µL was injected into an Agilent AdvanceBio Peptide Map (2.1 × 150 mm, 2.7 µm). Each sample batch began and ended with blank runs, with additional wash sequences inserted periodically between samples. The peptides were analyzed in positive mode with an electrospray ion source set to 5500 V, a decluttering potential (DP) of 100 V, a curtain gas flow of 30 psi, nebulizer gas 1 (GS1) 12, and an interface heater at 450 °C. The mass spectrometer acquired full TOF-MS scans of 1.62 s over a mass range of 350–1800 *m*/*z*. Product ion MS/MS scans were performed over 50–1800 *m*/*z* production, exceeding a threshold of 100 counts, with charge states ranging from +2 to +5.

### 4.12. Protein Identification and Functional Annotation

Protein identification was conducted using PEAKS software (version 7.0; BSI, Toronto, Canada), utilizing the *Homo sapiens* reference genome database (UP000005640) containing 82,485 proteins. The raw data analysis incorporated de novo protein sequencing, database searches, and identification of specific post-translational modifications. A false discovery rate of 0.5% was established, with [−10×log(p)] calculated accordingly. The PEAKS parameters were set as follows: (i) 5 ppm for precursor ion mass tolerance; (ii) [50] 0.1 Da for fragment ion mass tolerance (error tolerance); (iii) tryptic enzyme specificity allowing three missed cleavages; (iv) monoisotopic precursor mass and fragment ion mass; (v) cysteine carbamidomethylation as a fixed modification; and (vi) variable modifications, including acetylation of lysine, deamidation of asparagine and glutamine, oxidation of methionine, and conversion of glutamic acid and glutamine to pyroglutamate. The mass spectrometry proteomics data have been deposited to the ProteomeXchange Consortium via the PRIDE partner repository with the dataset identifiers PXD061526 and 10.6019/PXD061526.

GO annotation was performed using the functional annotation module in the Omicsbox software (version 3.0.30). BLASTp search, GO mapping, and annotation were performed using default parameter settings (database, Nr; e-value cut-off = 10–5; lineage, mammals). From each pair-wise comparison dataset, the top 20 annotated GO terms in the molecular function and cellular component categories were visualized in word cloud format (word size represents the sequence count in each GO term).

### 4.13. Western Blot Analysis

After husk treatment, the colon cancer cells were collected and placed in a prepared cold lysis buffer (8 M Urea, 0.8 M NH_4_HCO_3_, and pH 8) containing protease inhibitors. Then, the cells were homogenized before a BCA Protein Assay Kit (Thermo Fisher Scientific, #23227) was used for concentration determination. The cell lysates (30 µg) were separated using sodium dodecyl sulfate–polyacrylamide gel electrophoresis (SDS-PAGE) and then transferred to a nitrocellulose membrane (0.2 µm, Bio-Rad Laboratories, Inc., Hercules, CA, USA). After blocking with 4% skim milk in PBS buffer for 1 h at room temperature, the membrane was incubated with the primary antibody overnight at 4 °C. The primary antibodies used were rabbit polyclonal anti-human MMP2 (#4022), MMP9 (#3852), N-cadherin (#4061), and GAPDH (#2118) (dilution 1:500; Cell Signaling Technology). Then, nitrocellulose membranes were washed with 0.1 M PBS and Tween 20 (PBST) three times. The membrane was incubated with the goat anti-rabbit IgG (H + L) secondary antibody-HRP conjugate for 1 h at room temperature and washed three times with PBST. Finally, the membrane was imaged using a chemiluminescence imaging instrument (ChemiDoc™ Imaging System #12003153, Bio-Rad Laboratories, Inc., Hercules, CA, USA) and quantified using Quantity One software (Bio-Rad Laboratories, Inc., USA).

### 4.14. Statistical Analysis

The experimental data are presented as the mean ± standard deviation (SD) and were analyzed using GraphPad Prism 7 software (version 7.04). Comparisons between the extracts and controls were conducted using *t*-tests and analysis of variance (ANOVA). The IC_50_ for cell viability was calculated from dose–response data using a non-linear regression model with four-parameter logistic (4PL) curve fitting. Differences were considered statistically significant at * *p* < 0.05, ** *p* < 0.01, and *** *p* < 0.001 for cell viability, cell migration, cell invasion, colony formation, and Western blotting. Moreover, transcriptomic and proteomic data were considered statistically significant at * p < 0.05.

## 5. Conclusions

In this study, we investigated the effects of a sacha inchi husk extract on colon cancer HCT116 and HT29 cells, which provided accumulated evidence that the extract possesses anticancer properties. We presented cellular and molecular components that are impacted. Phytochemical screening of the husk extract demonstrated high total phenolic and total flavonoid contents. The chemical of highest abundance was naringenin, followed by lidocaine. The combined outcomes of in vitro cell assays and molecular gene and protein analyses support the bioactivity of the extract’s compounds in the suppression of colon cancer cell migration and invasion and the inhibition of colony formation through the upstream (EGFR) and downstream (EMT) pathways in colon cancer. In addition, the cellular experiments showed promising anticancer effects; however, these clinical conclusions require further validation through in vivo studies and specific tests to confirm efficacy, safety, and mechanisms of action under more complex physiological conditions. Importantly, the extract has potential for development into a dietary supplement or medical product to help treat colon cancer. This study provides support for the anticancer activity of sacha inchi husk extracts, which could assist farmers in developing new economic opportunities from their crops and thereby increase the commercial value of *Plukenetia volubilis* in Thailand.

## Figures and Tables

**Table 1:** Summary of RNAseq analysis following cell line treatment with the husk extract.

Cell Line (Treatment Concentration)	Total DEGs	Significantly Upregulated	Significantly Downregulated
HCT116 (100 µg/mL extract)	15,589	7632	7957
HT29 (250 µg/mL extract)	15,566	7726	7840

**Table 2:** Bioactive phytocomponents identified in the ethanolic husk extract using LC-MS.

Compound	Class	Activity	Ref.
Alpha, beta-trehalose	Disaccharide	Targeting cell progression, angiogenesis, and metastasis pathways at molecular level and targeting EGFR, PI3K, Akt, VEGF, and MMP9 proteins inside the cell.	[30]
Vitamin B6	Pyridine	Reducing inflammation, cell proliferation, oxidative stress, and anti-colorectal cancer.	[24]
Trigonelline	Pyridine	Inhibiting the invasion of hepatoma cells (AH109A).	[31]
Kaempferol 7-O-glucoside	Flavonoid glycoside	Anti-tumor effect through cell cycle arrest and apoptotic induction in cervix carcinoma (HeLa cells).	[32]
Echinocystic acid 3-glucoside	Triterpenoid glycoside	Induces the apoptosis and inhibits the migration and invasion of non-small-cell lung cancer cells.	[33]
Ingenol	Diterpene ester	Inhibits the growth of pancreatic cancer cells in vitro via STING	[34]
Dihydrokaempferol	Flavonoid	Suppression of apoptotic activity in rheumatoid arthritis	[35]
Protoporphyrin IX	Porphyrin	Inhibition of oncogenic Ras/MEK significantly in vitro and in vivo (colon and breast cancer)	[36]
Naringenin	Flavonoid	Inhibits colorectal cancer cell growth by repressing the PI3K/AKT/mTOR signaling pathway	[29]
Luteolin	Flavonoid	Induction of apoptosis and inhibition of cell proliferation, metastasis, and angiogenesis.	[37]
Triphenyl phosphate	phenyl phosphate	Induces cell proliferation and migration ability in colorectal cancer.	[38]
Mono-2-ethylhexyl phthalate	phenolic	Progression of CRC through AKT-β-catenin signaling.	[39]
2-Hexyl-4-pentynoic acid	Carboxylic acid	A novel radiosensitizer to breast cancer cells through increasing the instability of DNA repair proteins.	[40]
Palmitamide	Fatty acid	Anticancer activities on lung cancer cell line (NCI-60).	[41]
Phenyl isothiocyanate	Phenolic	Inducing apoptosis in breast cancer (HER2)-expressing tumor cells in vitro and in vivo.	[42]
Diphenylamine	Phenylamine	Suppresses androgen receptor and protein in prostate cancer.	[43]
Phthalic anhydride	Phenyl anhydride	Inhibits pro-inflammatory mediators through suppression of STAT3 activation in mice.	[44]
Bomyl acetate	Ester	Anticancer activity in human gastric cancer (SGC-7901) cells by inducing apoptosis, DNA fragmentation, and G2/M cell cycle arrest.	[45]

## Data Availability

Raw data for whole transcriptome sequencing are deposited in the NCBI Bioproject and SRA databases under Bioproject accession number PRJNA1236814. Raw data for peptide mass spectrometry are deposited in the PRIDE database under accession number PXD061526.

## Supplementary Materials

The following supporting information can be downloaded at https://www.mdpi.com/article/10.3390/ijms262110514/s1.

## Abbreviations

The following abbreviations are used in this manuscript:

CES	Collision energy spread
DEGs	Differentially expressed genes
DMSO	Dimethyl Sulfoxide
DP	Decluttering potential
DTT	Dithiothreitol
EMT	Epithelial–Mesenchymal Transition
FA	Formic acid
GC-MS	Gas chromatography–mass spectrometry
GO	Gene Ontology
IAA	Alkylating reagent
LC-MS	Liquid Chromatography–Mass Spectrometry
MET	Mesenchymal–Epithelial Transition
MMP	Matrix metalloproteinases
NIST	National Institute of Standards and Technology
PBS	Phosphate-buffered saline
PBST	Phosphate-buffered saline and 20% Tween
RNAseq	RNA sequencing
PMSF	Phenyl-Methyl-Sulfonyl-Fluoride
QTOF	Quadrupole Time-of-Flight
SRA	Sequence Read Archive
SD	Standard deviation
SDS-PAGE	Sodium Dodecyl Sulfate–Polyacrylamide Gel Electrophoresis
TFC	Total flavonoid content
TPC	Total phenolic content
TIMPs	Tissue inhibitors of metalloproteinases
